# Effects of Impulsivity and Interpersonal Problems on Adolescent Depression: A Cross-Lagged Study

**DOI:** 10.3390/bs14010052

**Published:** 2024-01-15

**Authors:** Yanan Yang, Mingyangjia Tian, Yu Liu, Shaojie Qiu, Yuan Hu, Yang Yang, Chenxu Wang, Zhansheng Xu, Lin Lin

**Affiliations:** 1 Key Research Base of Humanities and Social Sciences of the Ministry of Education, Academy of Psychology and Behavior, Tianjin Normal University, Tianjin 300387, China; 2 Center of Cooperative Innovation for Assessment and Promotion of National Mental Health under Ministry of Education, Tianjin Normal University, Tianjin 300387, China; 3 Faculty of Psychology, Tianjin Normal University, Tianjin 300387, China; 4 Intelligent Laboratory of Child and Adolescent Mental Health and Crisis Intervention of Zhejiang Province, Zhejiang Normal University, Jinhua 321004, China; 5 Department of Psychology, Zhejiang Normal University, Jinhua 321004, China; 6 Faculty of Psychology, Beijing Normal University, Beijing 100875, China; 7 Collaborative Innovation Center of Assessment Toward Basic Education Quality, Beijing Normal University, Beijing 100875, China

**Keywords:** depression, impulsivity, interpersonal problems, adolescent, cross-lagged model

## Abstract

The dynamic changes over time in the relationships between impulsivity, interpersonal problems, and depression warrant further exploration. This study delves into the roles of impulsivity and interpersonal issues in the progression of adolescent depression over a year, using a sample of 271 Chinese adolescents (51.7% male, Mage = 12.60 ± 0.69). At three time points, impulsivity levels were assessed with the Chinese version of the Barratt Impulsiveness Scale-11, interpersonal problems with the Adolescent Self-Rating Life Events Check List, and depression with the Center for Epidemiologic Studies Depression Scale. Results revealed that both impulsivity and interpersonal problems serve as risk factors for depression, but the primary risk factor shifted over time. In early middle school stages, impulsivity was the predominant risk factor, while in later stages, interpersonal problems became the primary risk factor. These findings carry significant implications for directing prevention efforts and interventions for adolescent depression.

## 1. Introduction

Depression refers to an individual’s negative reactions to adverse situations in daily life and study, manifesting as a non-specific period of sadness, unhappiness, or gloominess [1]. Adolescence, a critical transitional phase marked by swift social, emotional, and cognitive development, sees a significant increase in the likelihood of depression compared to childhood [2]. According to the World Health Organization, depression has become the fourth largest disease in the world and is still growing rapidly [3]. Among Chinese adolescents, depression has emerged as one of the more prevalent mental disorders [4]. A comprehensive meta-analysis of 37 studies revealed that the overall prevalence of adolescent depression in 18 provinces/cities across China has peaked at an alarming 28.4% [5]. Depression severely hampers healthy adolescent development, detrimentally affecting academic achievement, social interactions, and personal growth. It can also lead to other mental illnesses and even suicidal ideation that threatens life safety [2,6,7,8]. The high prevalence and severe negative outcomes highlight the pressing need to bolster prevention and intervention strategies for adolescent depression. Exploring the risk factors and mechanisms of depression is a prerequisite for developing effective prevention and intervention.

Monroe and Simons proposed the diathesis-stress theory of depression, which considers both the individual’s internal predispositions and external stressors, providing a more integrative perspective for understanding depression [9]. In this theory, diathesis refers to a range of factors including cognition, personality traits, and coping styles, while stress encompasses diverse life events. The theory posits three potential relationships between diathesis and stress in the development of depression: firstly, depression may arise from the combined effects of both diathesis and stress; secondly, depression may be triggered solely by diathesis; and thirdly, depression may occur exclusively due to stress.

### 1.1. Impulsivity and Adolescent Depression

Impulsivity is a diathesis factor that has been widely emphasized by researchers. Impulsivity refers to individuals’ tendency to react rapidly and thoughtlessly to internal and external stimuli without considering the potential negative impacts of such reactions on themselves or others [10]. Impulsive individuals are more likely to overestimate the negative significance or underestimate the positive significance of things, manifesting overgeneralization, self-blame, rumination, etc., which are all related to depression [11]. It is therefore speculated that impulsive individuals are more prone to developing depression [12,13]. However, existing studies do not consistently support this view. A meta-analysis revealed mixed results from eighteen studies investigating the relationship between impulsivity and depression [14]: eleven studies reported higher impulsivity in the major depressive disorder group compared to healthy controls; four studies reported positive correlations between impulsivity and depression severity; one study revealed a negative correlation; and two studies found no association. These inconsistent findings could be attributed to the diverse subject pool, which included adolescents, middle-aged adults, and older adults from ten countries. Given that impulsivity is shaped by both genetic and environmental factors [15,16] and fluctuates over time [17], age differences could have contributed to the varied findings. Studies with samples of American and Chinese adolescents have shown that impulsivity shows different trends over time [18,19], suggesting that differences in the subject populations could yield different outcomes. Therefore, focusing on Chinese adolescents, this study examined the effects of impulsivity on depression across time, aiming to provide more nuanced evidence for prevention and intervention efforts.

### 1.2. Interpersonal Problems and Adolescent Depression

As previously noted, stress encompasses various life events, among which interpersonal problems warrant particular attention. Interpersonal problems refer to emotional connections formed through direct interactions between individuals [20]. Individuals with interpersonal problems have difficulties handling relationships with others [21]. For adolescents, interpersonal problems are closely tied to psychological development [22,23,24]. Positive interpersonal relationships provide individuals more channels for obtaining care and assistance, including emotional support, instrumental support, informational support, etc., which helps protect mental health [25]. In contrast, negative teacher-student and peer relationships increase adverse life experiences and hinder healthy psychological development [26]. According to the interpersonal risk model of depression, interpersonal problems are an important source of stress negatively affect emotions and foster depression [27]. A study in Iran using linear regression analysis found interpersonal problems could predict 21.4% of variance in depressive symptoms [28]. Moreover, given the strong emphasis on group harmony and cohesion in Chinese Confucian traditions, interpersonal problems undoubtedly exert considerable stress on Chinese adolescents. The China Mental Health Development Report (2019–2020) indicates that interpersonal problems significantly impact adolescent depression in China, with healthier interpersonal relationships correlating with lower depression levels [29]. Most of these findings are based on cross-sectional studies. However, upon entering middle school, adolescents’ interpersonal problems change markedly [30]. Adolescents experience rapid physical and mental development during their secondary school years. A study involving 11,743 secondary school students noted that in the seventh and eighth grades, physiological development rapidly matures, while psychological development transitions from a childlike state to maturity. This imbalance between physical and mental development can lead to a decline in the level of interpersonal communication [31]. As age increases, physical and mental development becomes more balanced in the ninth grade, and the level of interpersonal relationships rises. Interpersonal instability during this period may lead to changes in the relationship between interpersonal problems and depression. It is therefore necessary to examine the effect of interpersonal problems on Chinese adolescent depression over time and elucidate the mechanisms to inform prevention and intervention efforts targeting depression.

### 1.3. Longitudinal Effects of Impulsivity and Interpersonal Problems on Adolescent Depression

Although the effects of impulsivity and interpersonal problems on depression may vary over time, existing studies have mostly examined their individual impacts from a cross-sectional perspective, with limited research on how the two factors simultaneously affect Chinese adolescent depression as they change over time. The “Process-Person-Context-Time Model” (PPCT) integrates a more comprehensive perspective to explore individuals’ developmental processes [32], emphasizing that development occurs through interactions between personal characteristics and external activities. Based on this model, the relationship between risk factors and the development of depression in Chinese adolescents can be examined from a more comprehensive perspective. “Process” refers to the proximal processes which are progressively more complex reciprocal interactions between the developing person and the immediate microsystem over time that drive development. Interpersonal relationships occur in direct human contact, and the adolescent, as a party to the relationship, necessarily interacts with other people in the microsystem, so interpersonal problems belong to “process” factors in this study. “Person” refers to personal characteristics, to which impulsivity belongs, so impulsivity is a “personal” factor. “Context” refers to the environment in which an individual grows up, and from a macro perspective, it can refer to the cultural institutional system. The subjects of this study are all adolescents who grew up in China and are inevitably influenced by the Chinese social and cultural background. Therefore, the source of the subjects belongs to the “Environment” factor. “Time” refers to the ongoing influences of the above three factors on individual development. The present study followed adolescents for one year, which is a “time” factor. Therefore, within the framework of this model, the present study, rooted in the Chinese cultural context, integrates the effects of “process” and “personal” factors on individual development over time, namely how impulsivity and interpersonal problems change over time in relation to depression, in order to achieve an integrative understanding of the mechanisms underlying depression.

### 1.4. Current Study

In summary, a robust connection exists between impulsivity, interpersonal issues, and depression in adolescents. The levels of impulsivity and interpersonal problems change, particularly during adolescence, a phase marked by swift physical and mental development. Previous studies have not concurrently explored the effects of impulsivity and interpersonal problems on the progression of depression from a longitudinal standpoint. Therefore, based on the instability of impulsivity and interpersonal problems, this study constructs a cross-lagged model to determine how the relationships between impulsivity, interpersonal problems, and depression in Chinese adolescents change over time. Three hypotheses are proposed: (1) Impulsivity and interpersonal problems among Chinese adolescents are not stable, but vary over time. (2) Prior impulsivity and interpersonal problems predict subsequent depression levels. (3) The effects of impulsivity and interpersonal problems on depression transform over time.

## 2. Methods

### 2.1. Participants

The study adopted cluster sampling, selecting students in the 7th grade from a middle school in Tianjin, China as research subjects for longitudinal follow-up in 2017–2018. They administer the questionnaire three times in seventh and eighth grade, every six months. The first questionnaire survey had 271 adolescents complete the survey (140 males; age = 12.60 ± 0.69, range 10–15 years old). Due to reasons like absence and transferring schools, 40 and 20 students did not participate in the second, third surveys, respectively. Finally, 211 adolescents completed all 3 surveys (103 males; age = 13.62 ± 0.68, range 11–16 years old), with a follow-up rate of 91.34%. No significant differences existed on key observational variables between retained and lost subjects (*p* > 0.05). Demographic information of the participants is shown in Table 1.

### 2.2. Measures

#### 2.2.1. Chinese Version of Barratt Impulsiveness Scale-11

The Chinese version of Barratt Impulsiveness Scale-11 (BIS-11-CV), developed by Barratt and Patton [33] and revised by Zhou et al. [34], was used to assess impulsivity. The scale has 30 items divided into three subscales: cognitive impulsivity (e.g., “I like to think about complex problems”), motor impulsivity (e.g., “I act on impulse”), and non-planning impulsivity (e.g., “I plan for my future”). The scale uses a 4-point scoring system, from “never” to “always” scored 1–4 respectively, with some items reverse-scored. Higher scores indicate greater impulsivity. In the three surveys, the internal consistency coefficients were 0.893, 0.896 and 0.895 respectively.

#### 2.2.2. Adolescent Self-Rating Life Events Check List

The Adolescent Self-Rating Life Events Check List (ASLEC) developed by Liu et al. [35] was used to assess the impact of interpersonal problems. The scale has 27 items divided into 6 subscales, with the interpersonal relationship subscale having 5 items (e.g., “having conflicts with classmates or friends”). If the event did not occur in the participant’s life, it is scored 0; if it did occur, the impact of the event is rated from “no effect” to “very severe effect”, scored 1–5 respectively. Higher scores on the interpersonal problems subscale indicate greater impact of interpersonal problems on the individual. In the three surveys, the internal consistency coefficients for the interpersonal problems subscale were 0.794, 0.832 and 0.867 respectively.

#### 2.2.3. Center for Epidemiologic Studies Depression Scale

The Center for Epidemiologic Studies Depression Scale (CES-D) developed by Radloff [36] and revised by Chen et al. [37] was used to assess depression levels. The 20-item scale has four subscales: depressed affect (e.g., “I felt depressed”), positive affect (e.g., “I felt hopeful about the future”), somatic and retarded activity (e.g., “I had trouble keeping my mind on what I was doing”), and interpersonal (e.g., “I felt that people disliked me”). Items are scored on a 4-point scale from “did not occur” to “consistently occur”, scored 0–3 respectively, with some items reverse-scored. Higher total scores indicate higher depression levels. In the three surveys, the internal consistency coefficients were 0.884, 0.852 and 0.894 respectively.

### 2.3. Collection and Analysis of Data

This study was approved by the ethics committee of the Academy of Psychology and Behavior of Tianjin Normal University. Before each survey, consent was obtained from the school principal, class teacher, and students’ parents. Students voluntarily signed informed consent forms. All questions were answered by the students themselves. Psychology graduate students who received professional training served as proctors, distributing and collecting the questionnaires in the classroom after explaining the instructions. Students had the freedom to withdraw from the survey at any time.

Psychology graduate students conducted data entry and management. Descriptive statistics, correlation analysis, and common method bias tests were performed using SPSS 24.0. Cross-lagged model was constructed using Mplus 8.0. Model estimation adopted robust maximum likelihood estimation (MLR), and the model fit was evaluated using the following indicators: χ^2^/df, CFI, RMSEA, and SRMR. Good model fits were defined as CFI values of 0.95 and above, RMSEA values of 0.08 and below, and SRMR values of 0.08 and below [38,39].

## 3. Result

### 3.1. Common Method Bias Analysis

To test for common method bias, Harman’s single factor test was used [40]. (1) In the first measurement, there were 19 factors with initial eigenvalues greater than 1, and the variance explained by the first factor was 20.45%, which was less than 40%; (2) In the second measurement, there were 18 factors with initial eigenvalues greater than 1, and the variance explained by the first factor was 21.78%, which was less than 40%; (3) In the third measurement, there were 18 factors with initial eigenvalues greater than 1, and the variance explained by the first factor was 23.19%, which was less than 40%. In this study, there was no common method bias.

### 3.2. Descriptive Statistics and Correlation Analysis

For impulsivity, interpersonal issues, and depression at the three time periods, descriptive statistics and correlational analyses were performed. Table 2 shows a strong and positive relationship between the three variables for teenagers in terms of impulsivity, interpersonal issue stressors, and depressive levels.

### 3.3. Changes in Impulsivity and Interpersonal Problems across the Three Measurements

**Figure 1:**
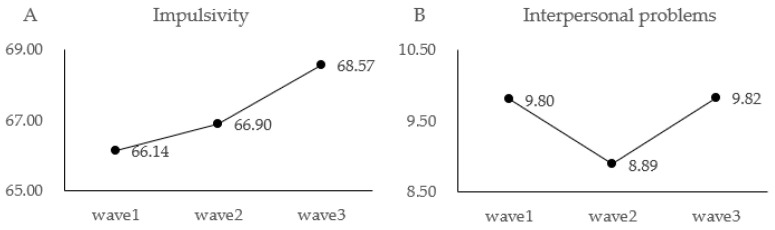
Changes in impulsivity and interpersonal problems across the three measurements.

The results of one-way repeated measures ANOVA with measurement time point as independent variable and impulsivity as dependent variable showed that the main effect of measurement time point on impulsivity was significant, F(2, 420) = 6.59, *p* < 0.01, η^2^p = 0.03; post hoc tests revealed that impulsivity at time point 3 was significantly higher than at time point 2 (*p* < 0.05), and impulsivity at time point 3 was significantly higher than at time point 1 (*p* < 0.01). See Figure 1A.

The results of one-way repeated measures ANOVA with measurement time point as independent variable and interpersonal problems as dependent variable showed that the main effect of measurement time point on interpersonal problems was significant, F(2, 420) = 3.29, *p* < 0.05, η^2^p = 0.02; post hoc tests revealed that interpersonal problems at time point 3 were higher than at time point 2, with marginal significance (*p* = 0.06). See Figure 1B.

### 3.4. Cross-Lagged Analysis of Impulsivity, Interpersonal Problems, and Depression

The relationship between impulsivity, interpersonal problems, and depression at the three time points was examined by constructing a series of competing models that correlated the error terms among the variables within the same time points. The results are shown in Table 3.

First, Model 1 estimated the stability of impulsivity, interpersonal problems, and depression across time, and the model fitting performed poorly. Model 2 added paths from impulsivity at the former time point to depression at the latter time point, and the model fitting performance was slightly improved (Δχ^2^ = 18.54, Δ*df* = 4, *p* < 0.01). Model 3 further added paths from depression at the former time point to impulsivity at the latter time point, and the model fitting performance achieved further improvement (Δχ^2^ = 14.50, Δ*df* = 4, *p* < 0.01). Model 4 achieved a better fit after adding paths from impulsivity and interpersonal problems at time point 1 to impulsivity and interpersonal problems at time point 3 (Δχ^2^ = 17.83, Δ*df* = 2, *p* < 0.001). Therefore, model 4 is the most acceptable of the four competitive models.

**Figure 2:**
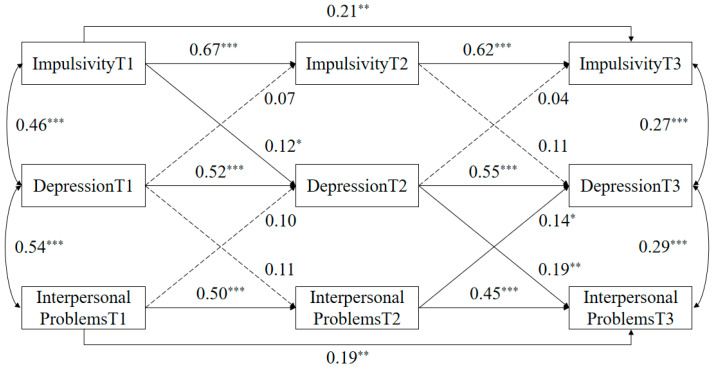
Cross-lagged model of impulsivity, interpersonal problems, and depression. * *p* < 0.05, ** *p* < 0.01, *** *p* < 0.001.

Based on Figure 2, the findings indicate that impulsivity at time point 1 positively predicts the level of depression at time point 2, suggesting that higher levels of impulsivity at the initial time point are associated with increased depression six months later. This highlights impulsivity as a risk factor for depression. On the other hand, there is a bidirectional relationship between interpersonal problems and depression at time points 2 and 3. Specifically, experiencing more interpersonal problems at time point 2 predicts greater severity of depression after six months. Similarly, higher levels of depression at time point 2 are associated with increased interpersonal problems at time point 3. However, it is worth noting that impulsivity at time point 2 does not predict depression at time point 3. This implies a shift in the risk factors for depression over time, with interpersonal problems becoming mocompared to impulsivity.

## 4. Discussion

This study conducted a one-year longitudinal investigation of Chinese adolescents to examine the interrelationships between impulsivity, interpersonal problems, and depression. We found that impulsivity and interpersonal problems were dynamic across the three measurements. In the first half year, impulsivity emerged as the primary risk factor for depression, while in the second half year, interpersonal problems took over as the predominant risk factor for depression. This indicates that the effects of impulsivity and interpersonal problems on depression change over time.

Numerous previous studies have shown that impulsivity is closely related to negative cognitions and difficulties with emotion regulation [41,42,43], which are crucial preconditions for depression [44,45,46]. Therefore, impulsivity is an important risk factor for depression, consistent with the results of this study. A characteristic feature of adolescence is the relative immaturity of self-control abilities [17]. Neurobiological studies indicate that the development and maturation of prefrontal cortical regions involved in impulse control and planning occur in early adulthood, with impulsivity levels gradually declining until stabilizing after the 20s [47]. The adolescents in this study had not reached this stage, and impulsivity exhibited an increasing trend across the three measurements, consistent with the immature development of the relevant cortical regions. Prior impulsivity significantly predicted depression levels six months later. Adolescents with higher impulsivity levels tend to harbor negative cognitions, weakening the positive effects of positive events and strengthening the negative effects of adverse events, resulting in more severe emotional distress and subsequent depression.

Interpersonal problems in adolescents initially decreased and then increased, with the change in the later stage being significant. Interpersonal problems also supplanted impulsivity as a key risk factor for depression during this stage. Establishing positive relationships with peers, teachers and parents facilitates adolescent development. However, during puberty, adolescents’ social networks expand continuously, increasing the likelihood of encountering problems like conflicts and rejection [48]. Additionally, traditional Confucian values place great emphasis on academic achievement [49]. Parents and society often view academic performance as the primary indicator of an individual’s success [50], making the transition to a prestigious high school through the middle school graduation exam a crucial milestone. From the 7th to 8th grade, adolescents enter a critical stage for laying the foundation for this exam. Academic competition among students intensifies, and pressure from teachers and parents mounts, which can also increase interpersonal conflicts. If not handled properly, these interpersonal problems become huge stressors leading to adolescent maladjustment [51], manifesting as increased depression levels six months later in this study. Therefore, paying attention to adolescents’ interpersonal problems is imperative.

The findings of this study provide richer empirical support for the diathesis-stress model and interpersonal risk model of depression. Interpersonal problems as a stressor and impulsivity as a diathesis can independently lead to depression, and their co-occurrence is not a prerequisite. The relationships between interpersonal problems, impulsivity and depression are dynamic, with different risk factors at different developmental stages of individuals. Identifying risks early and reducing the likelihood of maladjustment in adolescents is an important issue. Based on the results of this study, helping adolescents improve self-control and interpersonal skills at appropriate times are effective ways to reduce adolescent depression. Schools, communities, and families can take relevant measures. For schools, teaching knowledge and skills related to impulse control in the seventh grade would be beneficial when arranging mental health education curricula. In the eighth grade, the focus could be on interpersonal relationship management, paying attention to building positive peer, teacher-student, and family relationships, and handling conflicts and rejection. By providing students with necessary guidance to reduce their exposure to negative risks, their overall mental health can be promoted. In community mental health services, offering courses or workshops emphasizing building positive family relationships and guiding parents on managing children’s impulsive behaviors would be highly valuable. Through the collaborative efforts of schools, communities and parents, adolescents can be helped to withstand the risks of depression and supported in their healthy development.

The present study acknowledged several limitations that should be addressed in future research. Firstly, the sampling of students from only one secondary school limited the generalizability of the findings. Future studies could include participants from multiple schools or diverse educational settings. Secondly, this study relied solely on self-report questionnaires as the research method. Future research could employ additional research methods, such as observational or experimental designs, to complement the self-report data. By utilizing multiple sources of data, a more comprehensive understanding of the factors associated with depression in adolescents can be obtained. Furthermore, self-report measures might be subject to response biases and subjective interpretations. Future studies could consider incorporating objective assessments or obtaining data from multiple informants, such as parents, teachers, or mental health professionals, to provide a more comprehensive and well-rounded understanding of adolescent depression. In conclusion, future research should aim to expand the sample size, utilize diverse sampling strategies, employ multiple research methods, and incorporate data from various sources to further investigate the mechanisms underlying the factors associated with depression in adolescents and to enhance the validity and generalizability of the findings.

## 5. Conclusions

Impulsivity and interpersonal problems among Chinese adolescents changed over time within one year.

Prior impulsivity and academic performance predicted subsequent depression levels.

The effect of impulsivity and interpersonal problems on depression transformed over time. Impulsivity was a risk factor for depression in early middle school stages, while interpersonal problems was a risk factor in later middle school stages.

## Figures and Tables

**Table 1:** Demographic characteristics of participants (n = 211).

Demographic Characteristic		n	%
Family type	Nuclear family	157	74.41
	Extended family	40	18.96
	Not living with parents	6	2.84
	Single parent family	8	3.79
Only child	Yes	65	30.81
	No	145	68.72
	Missing	1	0.47
Family location	City	77	36.49
	County	25	11.85
	Town	100	47.39
	Village	6	2.84
	Missing	3	1.42
Family income	Very high	1	0.47
	Relatively high	43	20.38
	Average	144	68.25
	Relatively low	3	1.42
	Very low	0	0.00

**Table 2:** Descriptive statistics and correlation coefficients.

	1	2	3	4	5	6	7	8	9
1 Impulsivity T1	—								
2 Impulsivity T2	0.70 ***	—							
3 Impulsivity T3	0.66 ***	0.78 ***	—						
4 Interpersonal Problems T1	0.31 ***	0.30 ***	0.24 ***	—					
5 Interpersonal Problems T2	0.27 ***	0.30 ***	0.23 **	0.56 ***	—				
6 Interpersonal Problems T3	0.28 ***	0.29 ***	0.33 ***	0.52 ***	0.63 ***	—			
7 Depression T1	0.46 ***	0.38 ***	0.35 ***	0.54 ***	0.37 ***	0.38 ***	—		
8 Depression T2	0.39 ***	0.47 ***	0.41 ***	0.43 ***	0.50 ***	0.48 ***	0.63 ***	—	
9 Depression T3	0.33 ***	0.41 ***	0.46 ***	0.40 ***	0.42 ***	0.54 ***	0.52 ***	0.66 ***	—
M	66.14	66.90	68.57	9.80	8.89	9.82	15.16	16.26	15.58
SD	13.50	13.05	12.59	6.28	6.54	6.65	10.59	9.76	10.16

Note. ** *p* < 0.01, *** *p* < 0.001.

**Table 3:** Model fitting index.

Model	χ^2^/*df*	CFI	RMSEA	SRMR	Δχ^2^	Δ*df*	Correction
M1	89.67/25	0.91	0.11	0.13	—	—	1.13
M2	71.36/21	0.93	0.11	0.10	18.48 **	4	1.14
M3	56.72/17	0.95	0.11	0.06	14.58 **	4	1.12
M4	37.50/15	0.97	0.08	0.05	17.84 ***	2	1.11

Note. M1 is the stability model, M2 is the risk factor-driven model, M3 is the correlation model, and M4 is the correlation correction model, Δχ^2^ is the Satorra-Bentler chi-squared difference test. ** *p* < 0.01, *** *p* < 0.001. Age and gender as control variables in all models.

## Data Availability

The data presented in this study are available on request from the corresponding author. The data are not publicly available due to privacy and ethical restrictions.
